# Cannabis-Induced Malignant Catatonia: A Medical Emergency and Review of Prior Case Series

**DOI:** 10.7759/cureus.17490

**Published:** 2021-08-27

**Authors:** Batool Sheikh, Tinu Hirachan, Kunal Gandhi, Saral Desai, Rimsha Arif, Oleg Isakov

**Affiliations:** 1 Department of Psychiatry, Brookdale University Hospital Medical Center, Brooklyn, USA; 2 Department of Psychiatry, Saba University School of Medicine, Brooklyn, USA

**Keywords:** bush-francis catatonia rating scale, malignant catatonia, subtypes of catatonia, cannabis use, marijuana use, addiction psychiatry, psychiatry, substance use disorder (sud)

## Abstract

Few case reports of catatonia associated with cannabis use are reported. Here, we describe a case of a 35-year-old African American male who developed malignant catatonia following heavy cannabis use. The patient was brought to the emergency department (ED) for altered mental status, hypertension, and erratic behavior. Before his ED presentation, he was smoking cannabis in heavy amounts, confirmed by positive urine toxicology in ED. Initial lab results showed leukocytosis, elevated creatine phosphokinase (CPK) levels. Head CT scans without contrast, including cerebrospinal fluid (CSF) analysis, were nonsignificant. In ED, the patient was agitated, combative, mute, and rigid. He was sedated using 2 mg of intramuscular (IM) midazolam. Psychiatric consultation services suspected catatonia, and the patient scored 12 points on Bush-Francis Catatonia Rating Scale (BFCRS). Although the patient’s symptoms responded to 2 mg of IM lorazepam, the patient later relapsed, became tachycardic with blood pressure fluctuations, and his repeat BFCRS score was 18. At this point, the patient was diagnosed as having malignant catatonia, and his lorazepam dosage was increased up to 6 mg IM per day. After a few days of waxing and waning of his symptoms, he finally started to show constant improvement and gradually reduced his symptoms. Our case highlights the first-ever reported case of malignant catatonia associated with cannabis use.

## Introduction

Catatonia is diagnosed by the Diagnostic and Statistical Manual of Mental Disorders, 5th Edition (DSM-5) when three or more of the following symptoms are present: mutism, stupor, catalepsy, waxy flexibility, negativism, posturing, mannerism, stereotypy, unresponsive agitation, grimacing, echolalia, and echopraxia [1]. Malignant catatonia is a potentially lethal subtype of catatonia that presents with additional features of fever, autonomic instability, altered mental status, and elevated creatine phosphokinase (CPK) levels [2]. Catatonia is believed to be a neuro-psychomotor syndrome observed in any acutely ill patient with mental health conditions [3,4]. However, catatonia can also be observed with medical conditions, which are often overlooked [3,4]. The exact pathophysiology of catatonia remains unknown.

According to the National Survey on Drug Use and Health (NSDUH) 2016 results, marijuana is the most commonly used recreational drug in the United States, following tobacco and alcohol [5]. There are various ways to consume cannabis products such as “buds” (dried cannabis flowers), oil (butane honey oil, wax, crumble), and resin (hashish, bubble hash). These can be consumed either through smoking or as edible. Different products have different potencies that depend on the concentration of Δ9 tetrahydrocannabinol (THC), the primary psychoactive component of the cannabis plant [6].

Current literature suggests that cannabis use is associated with an increased risk of psychosis in susceptible individuals [7]. Cannabis as an etiology for developing catatonia is not well studied. However, a few case reports have reported catatonia linked to cannabis use. In this case study, we describe the first-ever reported case of malignant catatonia associated with cannabis use in a 35-year-old African American male.

## Case presentation

A 35-year-old African American male with a medical history of cannabis use disorder, opioid use disorder, major depressive disorder (MDD), and suspected history of schizophrenia as per documentation and hypertension was brought to the emergency department (ED) for altered mental status, bizarre and erratic behavior. According to the emergency medical service (EMS) personnel, the patient started behaving erratically at his friend’s house after smoking an unknown substance in heavy amounts. On his way to the hospital, the patient was selectively mute, tachycardic (137 bpm), and had a 220/110 mmHg blood pressure.

In the medical ED, the patient was diaphoretic, tremulous, tachycardic (134 bpm), and hypertensive (218/112 mmHg). He was agitated, physically combative, restless, and selectively mute. On examination, bilateral rigidity in the upper and lower extremities was noted. The patient was sedated using 2 mg of intramuscular (IM) midazolam as the patient displayed agitated behavior in the ED. Preliminary laboratories showed a leukocyte count (WBC) of 15,500/mm^3^, and CPK levels were 746 units/L. Urinalysis was unremarkable. Serum blood alcohol level was within the normal range. Urine toxicology was positive for only cannabinoids. A head CT scan without contrast did not reveal any abnormalities. His cerebrospinal fluid analysis was negative for any infectious etiologies that could explain his symptoms. Due to his past psychiatric history and negative workup for infectious and metabolic diseases, psychiatric consultation was obtained to assess an underlying psychiatric diagnosis that could explain his current presentation.

Upon psychiatric evaluation, the patient was suspected of having catatonia due to his waxy flexibility, mutism, rigidity, and periodic agitation. He scored 12 points on Bush-Francis Catatonia Rating Scale (BFCRS), and it was recommended that the patient receive an immediate dose of 2 mg IM lorazepam to assess if the patient’s catatonic symptoms improved. After the administration of 2mg of lorazepam in addition to antihypertensives and intravenous fluids, his vital signs normalized. At this moment, the patient was deemed medically cleared and transferred to the Comprehensive Psychiatric Emergency Program (CPEP) for further behavioral evaluation.

In the CPEP, the patient was on constant observation. He received a total of 6 mg IM lorazepam. Despite showing some initial improvements in his catatonic symptoms, the patient continued to deteriorate. His repeat BFCRS score increased to 18 points. The patient’s behavioral presentation did not remit, and soon he became tachycardic (136 bpm) with unstable, fluctuating blood pressure ranging from systolic 135 to 210 mmHg and diastolic 80 to 110 mmHg. At this moment, malignant catatonia was suspected, and further lab work was ordered to confirm the diagnosis. Repeated CPK showed an upward trend compared to his previous CPK levels of 746 units/L upon arrival in the ED, now trending >1000 units/L.

Due to his extreme fluctuations in vitals and increasing CPK levels, the patient was transferred back to the medical ED for stabilization. The patient was started on antihypertensive medication and eventually admitted to the medical floor for persistent altered mental status and non-traumatic rhabdomyolysis. The psychiatry team continued to follow the patient on the medical floor as the patient continued to show persistent symptoms of catatonia. The patient was continued on 2 mg IM lorazepam every 12 hours, which was titrated up to 4 mg IM by the recommendation of the neurology team. The psychiatry consult team also recommended increasing the frequency of the lorazepam dosing for better management of catatonic symptoms. An MRI of the brain was obtained at this moment, which did not show any abnormalities. After several days, the patient’s laboratory values normalized.

The patient was followed closely by the neurology and psychiatry consultation services. He received intravenous fluids, lorazepam, and quetiapine for symptoms of catatonia and substance-induced psychosis, as well as elevated CPK levels. The patient health improved significantly. Lab results showed a resolution of rhabdomyolysis and acute kidney injury, and his vital signs normalized. The patient’s catatonic symptoms resolved, and during the hospital course, the patient did not display acute disorganization of thought processes, speech, or behavior. Therefore, a working diagnosis of malignant catatonia secondary to cannabis use was proffered. The patient was monitored for several more days on the regular medical floor, his physical and behavioral symptoms were improved, and he was discharged.

## Discussion

The survey from 2016 to 2017 found that more than 39 million respondents used cannabis last year [8]. It was also found that adults with medical conditions were significantly more likely to use marijuana. Smoking was the preferred method of administration for most users (77.5%) [8]. Recently, there has been an upward trend in using recreational synthetic cannabinoids. These synthetic cannabinoids with street names including “Spice” and “K2” may be up to 100-fold more potent than THC and have been linked to increased risk of adverse psychiatric and medical consequences [9]. The increased popularity of these products recently is due to their low cost, easy availability, and lack of detection in drug screening [9].

The prevalence of catatonia is mainly studied in acutely ill psychiatric patients in an inpatient setting, ranging from 9 to 18% [10]. Catatonia is commonly observed in patients with delirium, mood disorders, schizophrenia, post-traumatic stress disorder (PTSD), and dissociative states. In the United States, emergency room visits related to cannabinoids are steadily increasing [11]. In the United States, ED visits for cannabis-only intoxication have increased more than 43% over seven years, along with a significant increase in adolescent-specific patients [12]. Recent trends have also indicated an uptick in emergency room and inpatient psychiatric unit admissions due to abnormal behavior secondary to synthetic cannabinoid intoxication [13]. Of patients who visited the ED in association with synthetic cannabinoid intoxication, less than 25% of patients were admitted for medical treatment, psychiatric treatment, or the ICU [13]. However, the association between cannabis and catatonia is less studied and less understood. Very little is known about this association and primarily through case reports. These case reports have been summarized in Table 1 [14-17].

**Table 1:** Comparison of case reports on cannabis use and catatonia BFCRS: Bush-Francis Catatonia Rating Scale

Case Reports	Mekala et al. [14]	Mekala et al. [14]	Manning et al. [15]	Caudron et al. [16]	Smith et al. [17]
Year reported	2020	2020	2020	2015	2014
Age	22	33	20	32	17
Gender	Female	Female	Male	Male	Male
Medical comorbidities	N/A	N/A	N/A	N/A	N/A
Psychiatric diagnoses	N/A	Schizophrenia	N/A	N/A	N/A
Cannabis (Type)	Smoking marijuana joints	Smoking marijuana joints	Vaping cannabis oil for two weeks duration	Smoking marijuana joints	Smoking synthetic cannabinoids; 2-3 grams gaily for two months duration
BFCRS score	N/A	N/A	19	39	N/A
Treatment	Lorazepam, duloxetine, and olanzapine	Lorazepam and olanzapine	Lorazepam	Lorazepam and memantine	Lorazepam and olanzapine electroconvulsive therapy (ECT)

The exact neurobiology and pathophysiology of catatonia remain unknown. However, there are indirect pieces of evidence to suggest the implication of several neurotransmitters. For example, lorazepam, which acts as gamma-aminobutyric acid (GABA)-A receptor agonist, improves symptoms of catatonia while flumazenil, a GABA antagonist, produces relapse of catatonia in patients treated with lorazepam [18]. Phencyclidine (PCP) and Cerestat (Cambridge Neuroscience, the UK) act as potent N-methyl-D-Aspartate (NMDA) glutamate receptor antagonists and produce catatonia [18]. Both GABA and glutamate play an essential role in the motor pathway of the brain, modulating the release of other neurotransmitters such as dopamine. The principal active component THC acts on cannabinoid receptor type 1 (CB1) receptors, which are G protein-mediated receptors. These receptors are found in high density in the cerebral cortex (mainly frontal lobe), basal ganglia, hippocampus, anterior cingulate gyrus, and cerebellum. The cannabinoids exert their effects by modulating neurotransmitters (including GABA, dopamine, or glutamate) released via the activation of presynaptic CB1 receptors [19]. THC works on CB1 receptors located on GABAergic neurons, inhibiting GABA release into synapses [20]. Such a decrease in GABA release might result in hypoactivity at GABA A receptor that could produce catatonia. Individual variance in CB1 receptor expression could explain why some individuals are more prone to developing catatonia from cannabis use [21]. Thus, there is some biological evidence to suggest the role of cannabis in catatonia, but further research needs to be done.

Benzodiazepines are currently considered the first-line treatment for any form of catatonia, including catatonia secondary to recreational substances such as cannabinoids. The exact mechanism of action is unknown, although it is theorized that the treatment of symptoms is related to the upregulation of GABA receptors, particularly in the orbitofrontal cortex. Downregulation of NMDA receptor activity via benzodiazepine has also been theorized. It is recommended to continue the exact benzodiazepine dosage once the patient’s catatonic symptoms respond sufficiently. Electroconvulsive therapy (ECT) should be considered for catatonic patients not responding to sufficient trials of benzodiazepines. Once the catatonia is resolved, patients can be started on antipsychotics if needed as they may no longer be at increased risk of developing neuroleptic malignant syndrome (NMS) due to catatonia [4].

## Conclusions

Cannabis is one of the most common recreational drugs used today and may be associated with adverse psychiatric reactions such as psychosis, depression, and anxiety. As seen in our case study, cannabis use may lead to catatonia, including malignant catatonia in susceptible individuals. Further research must examine the neurological association between cannabis use and catatonia. Treatment options for cannabis-induced malignant catatonia must also be examined to determine whether different management is required for treating catatonia due to cannabis use.
